# Knowledge and attitudes about end-of-life decisions, good death and principles of medical ethics among doctors in tertiary care hospitals in Sri Lanka: a cross-sectional study

**DOI:** 10.1186/s12910-021-00631-5

**Published:** 2021-05-26

**Authors:** Thashi Chang, Saumya Darshani, Pavithra Manikavasagam, Carukshi Arambepola

**Affiliations:** 1 Department of Clinical Medicine, Faculty of Medicine, University of Colombo, 25, Kynsey Road, Colombo, 00800 Sri Lanka; 2 Department of Community Medicine, Faculty of Medicine, University of Colombo, Colombo, Sri Lanka

**Keywords:** End-of-life, Good death, Ethics, Sri Lanka

## Abstract

**Background:**

Competent end-of-life care is an essential component of total health care provision, but evidence suggests that it is often deficient. This study aimed to evaluate the knowledge and attitudes about key end-of-life issues and principles of good death among doctors in clinical settings.

**Methods:**

A cross-sectional study was conducted among allopathic medical doctors working in in-ward clinical settings of tertiary care hospitals in Sri Lanka using a self-administered questionnaire with open- and close-ended questions as well as hypothetical clinical scenarios. Univariate and logistic regression analysis were used to identify the independent factors associated with knowledge and attitudes.

**Results:**

Of the responders who had not been a caregiver for a terminally ill relative (n = 390), 57.9% were men with a mean age of 36.5 years (SD = 8.2). Compared to undergraduate (65.6%; n = 256), only 27.4% (n = 107) had received end-of-life care training at postgraduate level. Only 65.9% of doctors favoured disclosing terminal prognosis to patients; 27.7% of doctors were aware of advance directives; 14.6% were aware of the correct time of death when certifying brain death; 70.3% felt more comfortable in withholding than withdrawing life-sustaining treatment; 61.3% were aware of do-not-attempt cardiopulmonary resuscitation (DNACPR) decisions while 26.7% felt reluctant to administer it; 15.1% thought that all life-sustaining therapy should be withdrawn with a DNACPR decision; and only17.9% were able to name the four principles of medical ethics while 57.9% could not name a single. Participants scored a mean of 9.2 (SD = 3.9) of a maximum 14 points when tested on principles of a ‘good death’. Doctors who had pursued postgraduate studies were more likely to be aware of breaking bad news (adjusted-Odds-Ratio:1.99; 95%CI = 1.19–3.32), advance directives (adjusted-OR: 4.15; 95%CI = 2.49–6.94), aware of certifying the correct time of death (adjusted-OR:2.37; 95%CI = 1.33–4.2) and less reluctant to make DNACPR decisions (adjusted-OR:1.74; 95%CI = 1.13–2.68). Doctors who had worked in ICU were more comfortable withholding than withdrawing treatment (adjusted-OR:1.99; 95%CI = 1.2–3.31).

**Conclusions:**

Knowledge and attitudes about end-of-life care, good death and principles of medical ethics among doctors in Sri Lanka were suboptimal. Structured training of end-of-life care needs to be integrated within curricula and in-service training.

**Supplementary Information:**

The online version contains supplementary material available at 10.1186/s12910-021-00631-5.

## Background

As much as possessing the competencies to provide care to treat illnesses and to maintain good quality health and longevity, doctors are expected to possess knowledge, skills and attitudes necessary to provide compassionate and appropriate care at the end-of-life. The need for adequate competencies in providing end-of-life care has been accentuated by the current global pandemic of COVID-19 that is claiming hundreds of thousands of lives irrespective of race, religion or creed.

An initial step in beginning a discussion on end-of-life care is the disclosure of a poor prognosis to the patient. Studies have shown that physicians frequently do not discuss prognosis with the patient or their caregiver, they present fewer facts and less detail concerning prognostic information compared with other types of information, do not adequately explore the patient’s understanding of his/her condition and are reluctant to provide a frank estimate of survival even if the patient requests this information [1–5]. In Sri Lanka, doctors commonly inform the family instead of the patient of a poor outcome of his or her illness despite evidence that patients prefer to be told whether they had a life-limiting illness and to discuss about prognosis and end-of-life decisions [6, 7].

Delivery of appropriate end-of-life care is dependent on whether doctors possess the requisite knowledge and attitudes apropos end-of-life issues, the concept of good death and the principles of medical ethics that guide decision-making in the last stages of the patient’s life which are often emotionally and ethically challenging. Thus, we conducted a study to assess the knowledge on the four principles of medical ethics, attitudes about communicating end-of-life decisions to patients, do-not-attempt cardiopulmonary resuscitation (DNACPR), advance directives, withholding and withdrawing life-sustaining therapy and good death, and their associated factors among doctors working in tertiary care hospitals in Sri Lanka.

## Methods

A cross-sectional study was conducted among allopathic medical doctors currently working in government tertiary care hospitals (teaching hospitals, provincial hospitals, district general hospitals) in Sri Lanka. At the time of the study, there were 6958 doctors employed in 34 tertiary-care hospitals distributed within the 25 administrative districts of Sri Lanka [8] and included doctors of all categories ranging from senior (consultant, senior registrar/chief resident) to junior (registrar/resident, senior house-officer) personnel. ‘Senior house-officer’ is the period of employment in a clinical setting without enrolment into any postgraduate course. Of the doctors working in tertiary care hospitals, intern house-officers who are at the beginning of their medical career and doctors who had not worked in an in-patient clinical setting (paediatric, medical, obstetrics or surgical wards or intensive care units) during the last one year were excluded from the study.

The sample of doctors to be recruited for the study from tertiary care hospitals was identified from a comprehensive register maintained of post-intern doctors working in government hospitals in Sri Lanka. This list is routinely updated and includes their current workstation and contact details including electronic mail addresses. The required sample size was 322 in order to detect an expected proportion of doctors having knowledge on the principles of medical ethics of 70.1% based on an Indian study among tertiary care doctors [9], alpha error of 1.96 and precision of 0.05. However, a number in excess to the calculated sample size (approximately tenfold of the required sample size, i.e., n = 3400) was randomly selected from the list of tertiary care hospital doctors in the register and invited to participate in the study. This was done because a high non-response rate was anticipated when data collection was not through face-to-face encounters [10] and because we were not aware as to how many on the register would fulfil the eligibility criteria including working in an in-ward clinical setting during the past one year.

Data were collected using a self-administered questionnaire, which included open- and close-ended questions as well as hypothetical clinical scenarios to elicit demographic characteristics, work and training experience, and knowledge and attitudes on end-of-life care, the core principles of medical ethics (autonomy, beneficence, non-maleficence and justice) [11], physician aid-in-dying and good death (Table 1 and Additional File 1: S1). Whether any of the participants had been caregivers for a terminally ill relative during the last one year was also recorded.

**Table 1:** Questions used in the survey (for the complete questionnaire, please see Additional File)

*On breaking bad news*
1. According to your knowledge, in a patient with advanced, progressive, incurable disease, with whom should the doctor discuss the diagnosis and prognosis? (Select one response only)
a. The patient only
b. The patient’s immediate family only
c. Both the patient and family
d. None
e. Other (please specify)
2. What are your attitudes on informing the patient about a diagnosis of terminal illness and its prognosis? (indicate your opinion of each statement as *Strongly agree; Agree; Disagree; or Strongly disagree*)
a. It will make the patient depressed
b. Is of no benefit to the patient
c. A grief reaction will occur, but the patient will adjust
d. It will reduce the patient’s anxiety associated with uncertainty
e. To know when death is coming is an essential prerequisite for a good death
f. The family (not the doctor) should break the news to the patient
*On advance directives*
3. Are you aware of advance directives (living wills)? (Select one response only)
a. No, I have never heard of it
b. I have heard, but not well aware of it
c. I am well aware of it
4. Can an attempted suicide (deliberate self-harm) be considered as an advance refusal of life-saving treatment? YES/NO
5. Would you transfuse blood in a patient in vascular shock due to active gastric bleeding and a haemoglobin of 4 g/dl, even if the patient has made an advance refusal of receiving any blood products? YES/NO
*On withdrawal and withholding life sustaining treatment*
6. A 28-year-old doctor with metastatic carcinoma has developed respiratory failure. She could live for several weeks if she is placed on a ventilator. Would you place her on a ventilator? YES/NO
7. A 28-year-old doctor who was ventilated following a road traffic accident has been confirmed to be brain dead. A 28-year-old man is in urgent need for a ventilator following deliberate self-harm with an insecticide. He could be saved if placed on a ventilator. There are no vacant ventilators available. Would you disconnect the doctor from the ventilator? YES/NO
8. What time would you record as the ‘time of death’ in a brain-dead patient who is disconnected from the ventilator?
9. Do you feel *more comfortable to withhold than to* withdraw life-sustaining therapy? YES/NO
*On ‘Do-not-attempt cardiopulmonary resuscitation (DNACPR)’ decisions*
10. Are you aware of ‘do-not-attempt cardiopulmonary resuscitation (DNACPR)’ decisions? (Select one response only)
a. No, I have never heard of it
b. I have heard, but not well aware of it
c. I am well aware of it
*If the answer is ‘a’ please skip question 11–15*
11. When would you consider a DNACPR order appropriate?
12. Who should make the DNACPR decision in an *unconscious* patient? (select one response only)
a. The medical team only
b. The family only
c. Both the medical team and family
d. Other (please specify):
13. Is it appropriate to withdraw all life sustaining therapy once a DNACPR decision has been made? YES/NO/DO NOT KNOW
14. Would you feel reluctant to make a DNACPR decision on a patient? YES/NO *If yes*, why?
15. Have you been involved in DNACPR decision? YES/NO
*On the concept of a ‘Good Death’*
16. Once ‘dying’ (end-of-life) has been diagnosed, who should take the *lead* role in ensuring that the patient has a good death? (Select one response only)
a. The caring physician
b. The family
c. A spiritual leader
d. Nursing staff
e. Other (please specify)
17. What would you consider as *essential characteristics of a good death*? (mark correct responses only)
1. To know when death is coming and to understand what can be expected
2. To be able to retain control of what happens
3. To be afforded dignity and privacy
4. To have control over pain and other symptom control
5. To have choice and control over where death occurs (at home or elsewhere)
6. To have access to information and expertise of whatever kind is necessary
7. To have access to any spiritual or emotional support required
8. To have access to hospice care in any location, not only in hospital
9. To have control over who is present and who shares the end
10. To have time to say goodbye, and control over other aspects of timing
11. To be able to leave when it is time to go, and not to have life prolonged pointlessly
12. To have lived a long life
13. To have lived a wholesome (virtuous) life
14. To be able to issue advance directives which ensure wishes are respected
*On medical ethics*
18. Name the four principles of medical ethics
19. Should physician aid-in-dying (which includes both ‘physician-assisted suicide’ and ‘euthanasia’) be legalized in Sri Lanka for patients with incurable, progressive and painful disease? YES/NO

The questionnaire was developed by an expert group comprising a Neurologist, Physician, Oncologist and a Clinical Psychologist. The questionnaire was pre-tested in a convenience sample of 10 tertiary-care hospital doctors representing the main four clinical specialities (medicine, surgery, paediatrics and obstetrics) who were not recruited for the study proper. The tool underwent judgemental validity to assesses whether the conceptual definitions have been appropriately converted into operational terms or not [12]. For this purpose, face validity was assessed by a non-expert group representing the target population; and content and consensual validity by an independent expert panel of relevant specialities. Each item of the tool was assessed for its relevance, appropriateness of the wording used and acceptability in the local context. Modified Delphi technique was used to achieve consensus among the experts following the ratings given for each item in the questionnaire.

The questionnaire was primarily administered via email using Google forms and the recipients were requested to complete it only if they qualified based on the stated eligibility criteria. This was complemented by sending a link to the Google form via social media and by posting a hard copy of the questionnaire with a self-addressed envelope to return the completed form. All participants were asked to complete the questionnaire only once, using any one of the preferred medium. Three reminders were sent by electronic and social media at two-week intervals, and not returning the form after three months was considered as either not willing to participate in the study or the doctor considering him/herself ineligible to participate in the study. Anonymity of the participants was ensured by not obtaining personal identification details in any of the forms and by using a newly created Google account that was discarded after collection of data. Returning the completed Google form was considered as implied consent of the participants. Prior to data collection, a pilot survey was conducted in a convenient sample of doctors to assess the feasibility and practical issues related to the Google form.

Ethics clearance was obtained from the Ethics Review Committee of the Faculty of Medicine, University of Colombo, Sri Lanka.

### Data analysis

Google form data were compiled into an Excel sheet, and all data were analysed using Statistical Package for Social Sciences (SPSS) version 20. The main analysis included data only of the doctors who had not cared for a relative with terminal illness during the preceding year. Data from those with a terminally ill relative were used only to compare the knowledge on the concept of ‘good death’ with that obtained by doctors included in the main analysis.

**Fig. 1:**
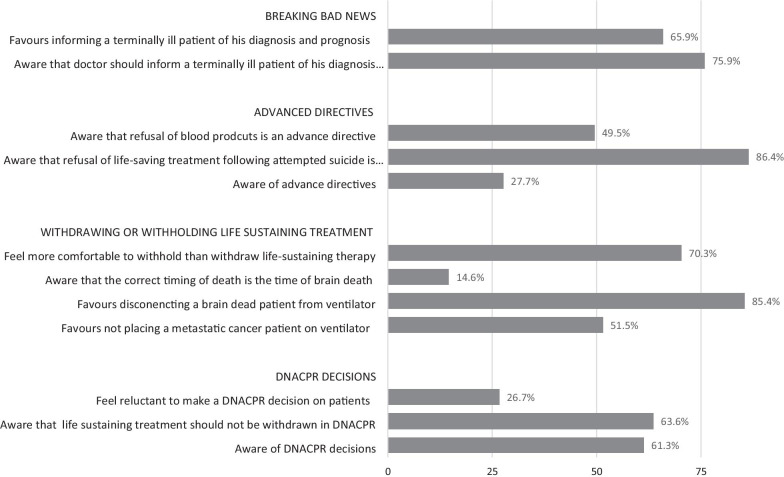
Selected knowledge and attitudes related to end-of-life care among doctors

The key to how responses to the questions were analyzed are given in Additional File 2: S2, which defines the terms used in Fig. 1 and Table 2.

**Table 2:** Factors determining knowledge and attitudes in end-of-life care (corresponding question numbers in the questionnaire are denoted as Q_x_ in the left column)

Characteristic	Gender		Done postgraduate		ICU work		Had in-service/PG EOL training		Years of work experience	
	Male 226	Female 164	Yes 162	No 228	Yes 122	No 268	Yes 107	No 283	< 15 years 291	≥ 15 99
*Breaking bad news*										
Knows that doctor should break bad news to patient Q1	175 77.4%	121 73.8%	131 80.9%	165 72.4%	90 73.8%	206 76.9%	86 80.4%	210 74.2%	233 80.1%	63 63.6%
Crude OR*	1.22 (0.76–1.95)		1.61 (0.99–2.63)		0.85 (0.52–1.39)		1.42 (0.82–2.46)		**2.29 (1.39–3.79)**	
Adjusted OR**	–		**1.99 (1.19–3.32)**		–		–		**2.71 (1.6–4.57)**	
Favours breaking bad news to terminally ill patient Q2	141 62.4%	116 70.7%	109 67.3%	148 64.9%	81 66.4%	176 65.7%	77 72.0%	180 63.6%	195 67.0%	62 62.6%
Crude OR*	0.69 (0.45–1.06)		1.1 (0.72–1.69)		1.0 (0.66–1.62)		1.47 (0.9–2.39)		1.21 (0.75–1.95)	
Adjusted OR**	–		–		–		–		–	
*Advance directives*										
Adequately aware of advance directives Q3	72 31.9%	36 22.0%	76 46.9%	32 14.0%	33 27.0%	75 28.0%	52 48.6%	56 19.8%	68 23.4%	40 40.4%
Crude OR*	**1.66 (1.05–2.64)**		**5.26 (3.33–9.09)**		0.95 (0.59–1.54)		**3.83 (2.37–6.19)**		**0.45 (0.28–0.73)**	
Adjusted OR**	–		**4.15 (2.49–6.94)**		–		**2.34 (1.39–3.94)**		–	
*Withholding/withdrawing life-sustaining treatment*										
Favours not placing a metastatic cancer patient on ventilator Q6	120 53.1%	81 49.4%	107 66.0%	94 41.2%	57 46.7%	144 53.7%	66 61.7%	135 47.7%	142 48.8%	59 59.6%
Crude OR*	1.16 (0.78–1.74)		**2.77 (1.83–4.22)**		0.75 (0.49–1.16)		**1.76 (1.12–2.78)**		0.65 (0.41–1.03)	
Adjusted OR**	–		**2.77 (1.83–4.22)**		–		–		–	
Favours disconnecting a brain-dead patient from ventilator Q7	194 85.8%	139 84.8%	132 81.5%	201 88.2%	96 78.7%	237 88.4%	85 79.4%	248 87.6%	249 85.6%	84 84.8%
Crude OR*	1.09 (0.62–1.92)		0.59 (0.34–1.04)		**0.48 (0.27–0.86)**		**0.55 (0.3–0.98)**		1.06 (0.56–2.01)	
Adjusted OR**	–		–		**0.49 (0.28–0.88)**		–		–	
Knows that time of death is the time of brain death Q8	38 16.8%	19 11.6%	34 21.0%	23 10.1%	22 18.0%	35 13.1%	20 18.7%	37 13.1%	43 14.8%	14 14.1%
Crude OR*	1.54 (0.85–2.79)		**2.37 (1.33–4.2)**		1.47 (0.82–2.62)		1.53 (0.84–2.78)		1.05 (0.55–2.02)	
Adjusted OR**	–		**2.37 (1.33–4.2)**		–		–		–	
More comfortable withholding than withdrawing ventilation Q9	160 70.8%	114 69.5%	118 72.8%	156 68.4%	97 79.5%	177 66.0%	81 75.7%	193 68.2%	206 70.8%	68 68.7%
Crude OR*	1.06 (0.69–1.65)		1.24 (0.79–1.93)		**1.99 (1.2–3.31)**		1.45 (0.87–2.41)		1.11 (0.67–1.81)	
Adjusted OR**	–		–		**1.99 (1.2–3.31)**		–		–	
*DNACPR*										
Adequately aware of DNACPR Q10	147 65.0%	88 53.7%	105 64.8%	130 57.0%	86 70.5%	149 55.6%	75 70.1%	160 56.5%	170 58.4%	65 65.7%
Crude OR*	**1.61 (1.07–2.42)**		1.39 (0.92–2.11)		**1.91 (1.21–3.02)**		**1.8 (1.12–2.9)**		0.74 (0.46–1.18)	
Adjusted OR**	–		–		**1.76 (1.11–2.81)**		–		–	
Aware when to consider DNACPR Q11	53 23.5%	32 19.5%	44 27.2%	41 18.0%	33 27.0%	52 19.4%	36 33.6%	49 17.3%	67 23.0%	18 18.2%
Crude OR*	1.26 (0.77–2.07)		1.7 (1.05–2.76)		1.54 (0.93–2.54)		**2.42 (1.46–4.02)**		1.35 (0.75–2.4)	
Adjusted OR**	–		–		–		**2.48 (1.49–4.14)**		–	
Aware that the medical team makes the DNACPR decision in an unconscious patient Q12	45 19.9%	31 18.9%	30 18.5%	46 20.2%	20 16.4%	56 20.9%	24 22.4%	52 18.4%	51 17.5%	25 25.3%
Crude OR*	1.07 (0.64–1.78)		0.89 (0.54–1.49)		0.74 (0.42–1.3)		1.29 (0.75–2.22)		0.63 (0.37–1.09)	
Adjusted OR**	–		–		–		–		–	
Knows that DNACPR does not entail withdrawing life-sustaining treatment Q13	152 67.3%	96 58.5%	107 66.0%	141 61.8%	89 73.0%	159 59.3%	78 72.9%	170 60.1%	190 65.3%	58 58.6%
Crude OR*	1.46 (0.96–2.21)		1.2 (0.79–1.83)		**1.85 (1.16–2.95)**		**1.79 (1.1–2.92)**		1.33 (0.83–2.12)	
Adjusted OR**	–		–		**1.74 (1.08–2.79)**		**1.84 (1.1–3.06)**		–	
No reluctance to make DNACPR decision Q14	153 67.7%	89 54.3%	114 70.4%	128 56.1%	81 66.4%	161 60.1%	77 72.0%	165 58.3%	181 62.2%	61 61.6%
Crude OR*	**1.77 (1.17–2.67)**		**1.86 (1.21–2.84)**		1.31 (0.84–2.05)		**1.83 (1.13–2.98)**		1.02 (0.64–1.64)	
Adjusted OR**	**1.64 (1.08–2.5)**		**1.74 (1.13–2.68)**		–		–		–	

*DNACPR* do-not-attempt cardiopulmonary resuscitation; *EOL* end-of-life; *ICU* intensive care unit; *PG* postgraduate

^*^Crude odds ratio (OR) calculated in univariate analysis on factors associated with knowledge and attitudes relevant to selected aspects of end-of-life care

^**^Adjusted odds ratio (OR) calculated in logistic regression analysis on factors associated with knowledge and attitudes relevant to selected aspects of end-of-life care after adjusting for confounders

For assessing knowledge on the concept of ‘good death’, participants were tested on 12 previously identified principles of a good death [13]. In addition to the 12 correct principles, 2 incorrect responses were included into the list of options to identify acquiescence bias [14]. One mark was allocated for each correct response, 0 for no answer and minus one mark for each incorrect response, giving a total score range of − 14 to + 14. This scoring system was designed for this study and had not been previously validated.

Data were summarised using mean and standard deviation (SD) for quantitative data, and proportions used for qualitative data. Further, the individual factors associated with knowledge and attitudes relevant to some important aspects of end-of-life care were assessed using univariate analysis, and thereafter in logistic regression analysis using backward LR method. In the models tested on each aspect, adjusted odds ratios (adj. OR) were calculated to ascertain the independent role of sex, religion, postgraduate training, ICU training, in-service/postgraduate training in end-of-life care and work experience (independent variables) on having adequate knowledge and favourable attitudes on the selected aspects (dependent).

## Results

The questionnaire was sent as a Google form to 3400 post-intern tertiary care hospital doctors who had email addresses in the register. Of them, 450 doctors who were eligible and consenting for the study responded from all 25 administrative districts in Sri Lanka. The response rate could not be accurately calculated since we were not aware how many of the 3400 that we invited to the study met the eligibility criteria. Among the responders, there were 60 doctors who had been family care-givers for at least one terminally ill first-degree relative and were excluded from the main analysis. The socio-demographic and professional training profile of the doctors in the main analysis (n = 390) is shown in Table 3. Their mean age was 36.5 years (SD = 8.2) with work experience on average of 10.5 years (SD = 8.4). The majority were junior level doctors (SHOs and Registrars/Residents). Three fourths had received formal training in end-of-life care during medical training. Compared to the undergraduate phase (65.6%), only a quarter (27.4%) had received such training at in-service or postgraduate (PG) level. However, only a third felt that they have had adequate training to feel confident in handling issues at the end-of-life. Small group discussions and role play were cited as the most preferred training methods.

**Table 3:** Characteristics and formal training in end-of-life care among doctors (N = 390)

Characteristics	No. (%)
**Current position**	
Senior house officer*	228 (58.5%)
Registrar/resident	66 (16.9%)
Senior registrar/chief resident	24 (6.2%)
Consultant	72 (18.5%)
**Males**	226 (57.9%)
**Have ever worked in an ICU setting**	122 (31.3%)
**Had undergraduate training in EOL care (n = 390)**	
Lectures	213 (54.6%)
Small group discussions	114 (29.2%)
Role play	57 (14.6%)
One or more of the above methods	256 (65.6%)
**Had in-service/postgraduate training in EOL care (n = 390)**	
Lectures	69 (17.7%)
Small group discussions	47 (12.1%)
Role play	25 (6.4%)
Formal training sessions during overseas training	10 (2.6%)
Local workshops/postgraduate course work	3 (0.8%)
One or more of the above methods	107 (27.4%)
**Either undergraduate or postgraduate or both**	297 (76.2%)
**EOL training perceived as adequate**	
Yes	123 (31.5%)
No	178 (45.6%)
Not certain	89 (22.8%)
**Most preferred training method on managing EOL issues**	
Lectures	49 (12.6%)
Small group discussions	98 (25.1%)
Role play	107 (27.4%)
Workshops/demonstrations	24 (6.2%)
Clinical exposure/experience	31 (7.9%)
Not certain	81 (20.8%)

*EOL* end-of-life; *ICU* intensive care unit

^*^Senior house-officer is the period of employment in a clinical setting immediately following internship and before enrolling into a postgraduate course

Figure 1 shows selected knowledge and attitudes related to end-of-life care among doctors in the study while Table 2 shows the factors associated with each of these aspects after adjusting for confounders using regression analysis.

### Breaking bad news

With regards to knowledge on breaking bad news, 75.9% of doctors were aware that the diagnosis and prognosis of advanced, progressive or incurable disease should be discussed with both the patient and family (Fig. 1); 3.8% would inform only the patient; 23.1% would inform only the family; and 1% would decide whom to inform based on the individual circumstances.

With regard to their attitudes on breaking bad news, the majority believed that it would reduce the patient’s anxiety associated with uncertainty (73.6%) and that it would enable the patient to adjust (88.5%). However, 84.6% of doctors also believed that it would make the patient depressed and 10.5% felt that it would be of no benefit to inform the patient. 11% felt that it should be left to the family to break bad news to the patient. Overall, 65.9% of the doctors favoured breaking bad news to patients (Fig. 1).

Postgraduate training (aOR = 1.99) and a shorter work experience (aOR = 2.71) were independently associated with adequate knowledge towards breaking bad news (Table 2).

### Advance directives (living wills)

Only 27.7% of doctors were aware of advance directives (Fig. 1). This was independently associated with postgraduate training (aOR = 4.15) as well as with end-of-life care training received at any time during their medical career (aOR = 2.34) (Table 2). When asked whether one would transfuse blood in a patient in vascular shock and a low haemoglobin, even if the patient has made an advance refusal of receiving any blood products, 49.5% responded ‘no’ while 47.4% responded ‘yes’. However, 86.4% knew that attempted suicide (deliberate self-harm) should not be considered as an advance refusal of life-saving treatment.

### Withdrawing and withholding life sustaining treatment

Only 14.6% were aware that the time of death is the time of certifying brain death in a patient on a ventilator (Fig. 1); 31.8% thought it was the time of disconnection from the ventilator; 18.7% thought it was the time the heart stops; and 6.9% did not know.

With regards to attitudes on life sustaining treatment, 51.5% of doctors responded that they would not place patients with metastatic carcinoma on a ventilator in order to prolong life for a few weeks, while 85.4% had no reservations in disconnecting a brain-dead patient from the ventilator. Overall, 70.3% responded that they felt more comfortable in withholding than withdrawing life sustaining treatment (Fig. 1).

Doctors with postgraduate training were more likely not to place terminally ill patients on ventilators (aOR = 2.77) and to correctly certify the time of death (aOR = 2.37) (Table 2) while ICU trained doctors were less likely to disconnect brain dead patients from ventilators (aOR = 0.49) (Table 2) and were more comfortable withholding than withdrawing ventilation (aOR = 1.99).

### DNACPR decisions

61.3% of doctors responded that they were adequately aware of DNACPR decisions (Fig. 1) while 4.4% had never heard of it. When asked about when it would be appropriate to consider a DNACPR decision, one correct response was given by 42.3% of doctors, two by 19.2% and three by only 2.6%. With regard to who makes the decision of DNACPR, 19.5% of the doctors responded that it should be decided by the medical team in an unconscious patient, while 11% of doctors believed that it was the family’s decision and 61.3% believed it to be a shared decision between the medical team and the family. Only 63.6% of the doctors were aware that DNACPR decisions did not imply withdrawal of life-sustaining treatment (Fig. 1), while 15.1% thought that all life-sustaining therapy should be withdrawn once a DNACPR decision was made and 13.6% were unsure whether to withdraw or not. Although 58.7% of doctors claimed to have been involved with the care of patients having a DNACPR decision, 26.7% felt reluctant to consider a DNACPR decision (Fig. 1). The three most cited themes for the reluctance were that it was ‘not humane’, ‘against our religious beliefs’ and ‘the senior doctor and not junior doctors who should make the decision’.

ICU training was independently associated with greater awareness of DNACPR decisions (aOR = 1.76) (Table 2). Having had EOL care training (aOR = 2.48) was independently associated with knowing when to consider DNACPR decisions. Doctors with ICU training were more likely to be aware that DNACPR decisions did not entail withdrawal of life-sustaining treatment while being male (aOR = 1.64) and postgraduate training (aOR = 1.74) were associated with less reluctance in making a DNACPR decision.

### Good death

**Fig. 2:**
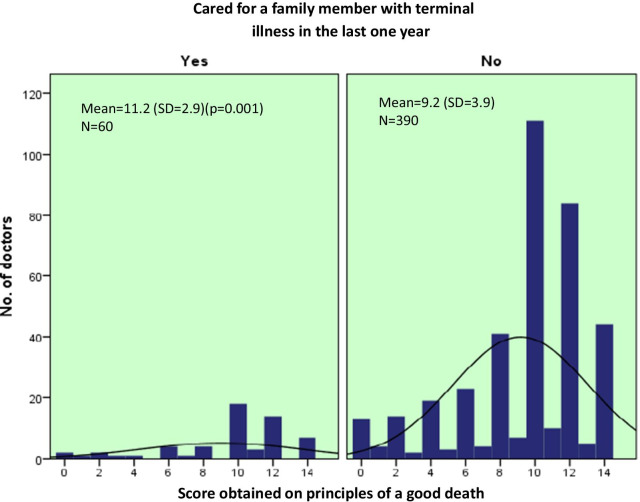
Good death knowledge score among doctors who have and have not cared for a relative with terminal illness

On evaluating the knowledge of the principles of a good death, 55.9% of doctors marked either one or both incorrect responses (items 12 and 13 of question 17 in Table 1) as correct. However, doctors scored a mean of 9.2 (SD = 3.9) of a maximum 14 points. In comparison, doctors who had been caregivers of a terminally ill relative scored significantly higher (mean = 11.2; SD = 2.9) (p = 0.001) (Fig. 2).

61% of doctors believed that it should be the caring physician who should take the lead role in ensuring that the patient has a good death; 17.7% thought it should be the family; 2.3% thought it should be a spiritual leader; 1% thought it should be the nursing staff; and 6.7% thought it should be all of the aforementioned categories.

Doctors with shorter service (aOR = 2.25; 95% CI = 1.37–3.7) and undergone postgraduate training (1.97; 95% CI = 1.23–3.16) were more likely to believe that the caring physician was responsible to ensure a good death at the end-of-life while Buddhists (aOR = 1.74; 95% CI = 1.09–2.76) were more knowledgeable about the principles of a good death independent of all other confounders.

### Principles of medical ethics and physician aid-in-dying

Only 17.9% of doctors were able to name all four principles of medical ethics (autonomy, beneficence, non-maleficence and justice), while 57.9% could not name a single. ‘Autonomy’ was the most (34.9%) while ‘justice’ was the least (25.9%) named ethical principle. 52.2% of doctors felt that physician aid-in-dying (which includes ‘physician-assisted suicide’ and ‘euthanasia’) should be legalized in Sri Lanka for patients with incurable, progressive and painful disease. None of the socio-demographic or professional characteristics of doctors were associated with favouring physician aid-in-dying. (analysis not shown).

## Discussion

Discussing end-of-life issues and death with patients and families continues to pose an uneasy challenge to most doctors leading to avoidance of such discussions often leaving patients and families unaware and unprepared, thus, losing the opportunity of a dignified end and a good death. The failure of doctors to discuss and to provide adequate guidance and support at the end-of-life has been attributed to multiple factors including prognostic uncertainty, fear of the impact on patients, navigating patient readiness and feeling inadequately trained for, or unaccustomed to, such discussions [1]. It is intuitive to assume that deficiencies in knowledge and attitudes related to end-of-life care, which stems from inadequate training and education in the formative years of doctors would be a significant contributor to this shortfall. This study explored the magnitude of such deficiencies and the factors that contributed to them in order to identify remediable measures. Despite having a low response rate and limited to a single South Asian population, this study provides some important insights highlighting the need and identifying measures likely to improve provision of end-of-life care, which is an area of medical practice that is often neglected.

In our study, knowledge and attitudes about end-of-life care, the concept of ‘good death’ and the principles of medical ethics among doctors practicing in tertiary care clinical settings in Sri Lanka were found to be lacking. Career progression to postgraduate training, having had in-service training in end-of-life care and training in ICU settings, and a shorter service duration were the most frequent independent determinants of better end-of-life care competencies. Doctors who had been caregivers for a terminally ill relative had a better knowledge of the principles of a ‘good death’.

The majority of doctors (n = 296; 75.6%) in our study were aware that it was necessary to disclose prognostic information, even of terminal illness, and felt that it would reduce the patient’s anxiety associated with uncertainty. However, many doctors also believed that disclosure could cause harm to patients by precipitating depression. This ambiguity probably has led to either the non-disclosure of such information or the practice of disclosing information to the family instead of the patient as was found to be preferred in 23.1% of our study participants and more (50%) in a previous study [7]. Although the reluctance to disclose prognostic information is common [1–5], the preference to disclose to the family instead of the patient may reflect the practice of collective decision making in certain cultures, as in ours, in contrast to the greater emphasis on individual autonomy as seen in Western cultures [1–3]. In such collective decision-making cultures, withholding information is ethically rationalised on the principle of beneficence based on the belief that disclosure causes more harm than good.

Advance directives (living wills) are legal documents that ensure that patient preferences regarding health, particularly regarding wishes for life-sustaining treatment, are respected when they are no longer able to make decisions for themselves because of illness or incapacity [15]. Advance directives are not legalised in Sri Lanka and this may explain the reason for its lack of awareness among doctors in our study. However, it is striking that almost half of the participants would transfuse blood in a patient when it’s a life saving measure even in the presence of an advance refusal. This may reflect the greater emphasis on beneficence and the lesser emphasis on the principle of autonomy among Sri Lankan doctors.

Although there is consensus in the medical, ethical, and legal communities that the withholding and withdrawing of life-sustaining treatment at the end-of-life are morally equivalent, there is a growing body of arguments challenging this assertion [16, 17]. However, withdrawal remains more emotionally challenging than withholding life-sustaining treatment as was also seen in our study. Notably, many doctors were not aware of determining the correct time of death when certifying brain death, which precludes justified withdrawal of ventilatory support.

Do-not-resuscitate orders were first reported in 1976 and are made when a patient has declined resuscitation, has a terminal prognosis, when cardiopulmonary resuscitation (CPR) is likely to be non-beneficial or if it is considered that the patient will not survive with sufficient quality of life [18]. In 2005 the term DNACPR replaced DNR to indicate that the order was limited to CPR and did not include pre-arrest life-sustaining interventions [19]. The DNACPR order is made by the caring physician in consultation with other members of the health care team and preferably after discussion with the patient, but the final decision remains the responsibility of the physician [20]. Although there is yet no specific legislature or guidelines on DNACPR in Sri Lanka, it is widely practiced applying the ethical principle of beneficence, when it is considered that administering CPR would not be in the best interest of the patient. In this context, it is significant that a notable proportion of our study participants were not aware of the DNACPR decision (38.7%) and its indications (35.9%).

Thus far, there is no universal definition of a good death. Although twelve guiding principles that define a ‘good death’ have been repeatedly identified in many studies, it is accepted that they should be applied respecting individual diversity in goals of care, perspectives, and preferences [13, 21, 22]. The relatively high knowledge score of the principles of a good death among the study participants is likely to be an overestimate due to a positive marking bias as reflected by over 50% marking the two incorrect responses as correct. As expected, doctors who had been caregivers for a relative with a terminal illness were better informed of the concept of a ‘good death’ [23]. In Sri Lanka, physician aid-in-dying is illegal. However, many doctors felt that it should be legalised for patients with chronic, incurable and progressive diseases resulting in unendurable pain.

In this study, postgraduate training, in-service training in end-of-life care and training in ICU settings were found to be independent determinants of better end-of-life care competencies. However, only about a quarter of our study population had received any form of postgraduate or in-service training in end-of-life care, thus reflecting the deficiencies in knowledge and attitudes related to end-of-life care. Inconsistency of structured end-of-life care curricula as an integral part of medical training, variability in the exposure to care of the dying and lack of opportunities to practice skills under supervision have been identified as universal causes of inadequacy of competencies in end-of-life care [24–26].

Studies on the influence of training interventions on patient and family outcomes are scarce [1]. Our study retrospectively identified education and training as independent determinants of better end-of-life care competencies. Future research should prospectively evaluate the impact of interventions such as in-service training and real-life encounters under supervision including palliative care consultations, on competencies in end-of-life care.

Some limitations should be considered when interpreting this study: the study population was limited to tertiary care doctors and the majority were at junior level, there was a wide variability of clinical disciplines from which the doctors were recruited that have variable exposure to end-of-life issues, the response rate to the survey was low and the inherent bias of postal surveys with only the inclined responding. Surveys do not necessarily reflect actual practice, but remains an acceptable approximate measurement. Although the findings may not be generalisable, this study adds important data to the literature of end-of-life care from a predominantly Buddhist Asian population. Furthermore, this study has succeeded in identifying the deficiencies in end-of-life care and through identification of associations, suggest remedial measures that are likely to be effective. The ones who did not return the Google form were either non-eligible doctors or non-responders. Thus, a non-response rate could not be ascertained in this study. Nevertheless, the minimum sample required of 322 was achieved in the study, with 450 returning completed Google forms out of the 3400 who were invited to the study. Previous studies have reported improved response rates with multimodality (postal plus electronic) surveys as replicated in our study and shown that the conclusions drawn from such surveys could be interpreted meaningfully with due recognition of the relatively low response rates [27, 28].

## Conclusions

Our study has identified significant deficiencies in knowledge and attitudes related to end-of-life care in Sri Lanka and highlights the need for mandatory training with simulations and real-life encounters under supervision in both undergraduate and in-service or postgraduate medical curricula. Application of the principles of medical ethics and good death should be adapted according to the social and cultural norms unique to the population. Normalising end-of-life discussions by embedding patient preferences at the end-of-life into routine history taking is likely to dissipate the anxiety that surround such discussions and enhance competent provision of end-of-life care.

## Supplementary Information

**Additional file 1: S1**. Questionnaire.

**Additional file 1: S2**. Key to analysis.

## Data Availability

All data generated or analysed during this study are included in this published article [and its Additional files].

## Abbreviations

aOR: Adjusted odds ratio
DNACPR: Do not attempt cardiopulmonary resuscitation

## References

[1] Brighton LJ, Bristowe K (2016). Communication in palliative care: talking about the end of life, before the end of life. Postgrad Med J.

[2] Hancock K, Clayton JM, Parker SM, der Wal S, Butow PN, Carrick S, Currow D, Ghersi D, Glare P, Hagerty R, Tattersall MH (2007). Truth-telling in discussing prognosis in advanced life-limiting illnesses: a systematic review. Palliat Med.

[3] Shahidi J (2010). Not telling the truth: circumstances leading to concealment of diagnosis and prognosis from cancer patients. Eur J Cancer Care (Engl).

[4] Lamont EB, Christakis NA (2001). Prognostic disclosure to patients with cancer near the end of life. Ann Intern Med.

[5] Kiely BE, Stockler MR (2019). Discussing prognosis, preferences, and end-of-life care in advanced cancer: we need to speak. JAMA Oncol.

[6] Pinto MVG, Varun R, Wanasinghe WMMPB, Jayasinghearachchi TMK, Herath HMTA, Kumarasiri PVR. A cross-sectional study of knowledge and attitudes of medical professionals towards end-of-life decisions in teaching hospitals of Kandy District (Sri Lanka). Anaesthesia Pain Intensive Care 2013;17(1):40–5.

[7] Perera M, Tennakoon T, Kumarasiri L, Jayasinghe S, Rathnayake R, Rajapaksha R (2013). Cancer in Sri Lanka: the question of, “to tell or not to tell”. Ceylon J Otolaryngol.

[8] Ministry of Health Sri Lanka. *Human Resource Profile. Health Staff Inposition by 31 December 2015*. Colombo: Health Information Unit & Management Development and Planning Unit, 2015.

[9] Singh S, Sharma PK, Bhandari B, Kaur R (2016). Knowledge, awareness and practice of ethics among doctors in tertiary care hospital. Indian J Pharmacol.

[10] Chang T, Ibrahim S, Ranasinghe HM (2020). Knowledge of stroke, its warning symptoms, risk factors and treatment among the general public and general practitioners in a south Asian population. J Stroke Cerebrovasc Dis.

[11] Beauchamp TL, Childress JF (1979). Principles of biomedical ethics.

[12] Abramson JH and Abramson ZH. Survey methods in community medicine: epidemiological research, programme evaluation, clinical trials. 5th ed. Churchill Livingstone, 1999.

[13] Smith R (2000). A good death. An important aim for health services and for us all. BMJ.

[14] Trakman GL, Forsyth A, Hoye R, Belski R (2017). Developing and validating a nutrition knowledge questionnaire: key methods and considerations. Public Health Nutr.

[15] Silveira MJ, Kim SY, Langa KM (2010). Advance directives and outcomes of surrogate decision making before death. N Engl J Med.

[16] Ursin LØ (2019). Withholding and withdrawing life-sustaining treatment: Ethically equivalent?. Am J Bioeth.

[17] Emmerich N, Gordijn B (2019). Beyond the equivalence thesis: how to think about the ethics of withdrawing and withholding life-saving medical treatment. Theor Med Bioeth.

[18] Burns JP, Truog RD (2016). The DNR order after 40 years. N Engl J Med..

[19] 2005 American Heart Association Guidelines for Cardiopulmonary Resuscitation and Emergency Cardiovascular Care. Circulation. 2005;112(24 Suppl):IV1–203. 10.1161/CIRCULATIONAHA.105.166550 16314375

[20] Pettersson M, Höglund AT, Hedström M (2018). Perspectives on the DNR decision process: A survey of nurses and physicians in hematology and oncology. PLoS ONE.

[21] Meier EA, Gallegos JV, Thomas LP, Depp CA, Irwin SA, Jeste DV (2016). Defining a good death (successful dying): literature review and a call for research and public dialogue. Am J Geriatr Psychiatry..

[22] Smith AK, Periyakoil VS (2018). Should we bury “the good death”?. J Am Geriatr Soc.

[23] Shi H, Shan B, Zheng J (2019). Knowledge and attitudes toward end-of-life care among community health care providers and its influencing factors in China: a cross-sectional study. Medicine (Baltimore).

[24] Schroder C, Heyland D, Jiang X, Rocker G, Dodek P (2009). Canadian researchers at the end of life network. Educating medical residents in end-of-life care: insights from a multicenter survey. J Palliat Med..

[25] Horowitz R, Gramling R, Quill T (2014). Palliative care education in U.S. medical schools. Med Educ..

[26] Hayley DC, Kalender-Rich JL, Mack J, Swagerty D (2018). Development of a hybrid simulated patient experience to practice care of the dying older adult. MedEdPORTAL..

[27] McMahon SR, Iwamoto M, Massoudi MS, Yusuf HR, Stevenson JM, David F, Chu SY, Pickering LK (2003). Comparison of e-mail, fax, and postal surveys of pediatricians. Pediatrics.

[28] Sur RL, Scales CD, Preminger GM, Dahm P (2006). Evidence-based medicine: a survey of American Urological Association members. J Urol.

