# Supplementary material for: Knowledge and attitudes about end-of-life decisions, good death and principles of medical ethics among doctors in tertiary care hospitals in Sri Lanka: a cross-sectional study
Source: BMC Med Ethics. 2021 May 26;22:66. doi: 10.1186/s12910-021-00631-5 (PMC8152188; doi:10.1186/s12910-021-00631-5)
Supplement: Supplementary file 1 — Additional file 1: S1. Questionnaire. [file 12910_2021_631_MOESM1_ESM.docx]

**Knowledge and attitudes of hospital doctors about end-of-life decisions and good death**

Dear Colleague,

Please fill this questionnaire only if you are currently working as a medical professional (excluding intern house officers) in an **in-patient hospital setting** (wards, ICU, Burns Unit, etc) including during the last one year. Please ignore this if you have already filled our online Questionnaire. The whole questionnaire takes **less than 10 minutes** to complete.

*The questions relate to* ***you****r experience and judgment. Please do not discuss the questions.* ***You*** *may not have experienced some of the scenarios described in the questionnaire. In such situations, indicate what* ***you*** *would do if faced with such a situation*.

Your contribution to this study is greatly appreciated.

Thanking you in advance,

Prof Thashi Chang

**Background information**

1. Age: ………………….Years

Male

Female

1. Gender:
2. What is your religion? .......................................................................
3. In which year did you graduate? ……………………………
4. In which ***type*** of hospital are you working currently?

.....................................................................................................................................

1. State the district your hospital belongs to. ……………………………………….

RHO

SHO/MO

Registrar

1. What is your current position?

Senior Registrar

Consultant

1. In which speciality are you currently working (eg: General Medicine, Orthopaedic, Anaesthesia, Burns unit etc.)?

..........................................................................

Yes

No

1. Have you ever worked in an intensive care unit for at least 6 months?

Yes

No

1. Have you had a family member with terminal illness in the last 6 months?
2. Have you had formal undergraduate teaching in handling end-of-life issues? **(Mark √, multiple responses are allowed)**

- None
- Lectures
- Small group discussion
- Role play
- Other

(Please specify):..........................

1. Have you had formal postgraduate teaching in handling end-of-life issues? **(Mark √, multiple responses are allowed)**

- Not done postgraduate
- None
- Lectures
- Small group discussion
- Role play
- Other(Please specify):............................................

1. Do you think that the above exposure (related to question 11 and 12) had prepared you *adequately* in handling the end-of-life issues that you have faced so far in your career?

Not had an exposure

Yes

No

1. Which teaching-learning activity do you think would best prepare you to face such situations?

………………………………………………………………………………………………………………………………………

**Questionnaire**

*End-of-life is defined as when the patient is likely to die within the next 12 months. This includes patients whose death is imminent (within a few hours or days) and patients with advanced, progressive, incurable disease.*Following are questions related to end-of-life issues.

***On Breaking Bad News***

1. According to your knowledge, in a patient with advanced, progressive, incurable disease, with whom should the doctor discuss the diagnosis and prognosis? **(Select one response only)**
2. The patient only
3. The patient’s immediate family only
4. Both the patient and family
5. None
6. Other (Please specify): ………………………...................……………
7. What are your attitudes on informing the patient about a diagnosis of terminal illness and its prognosis? (**Mark √ in the appropriate box)**

**SA-Strongly agree A- Agree D- Disagree SD- Strongly disagree**

|  |  | **SA** | **A** | **D** | **SD** |
| --- | --- | --- | --- | --- | --- |
| **a.** | It will make the patient depressed. |  |  |  |  |
| **b.** | Is of no benefit to the patient. |  |  |  |  |
| **c.** | A grief reaction will occur, but the patient will adjust. |  |  |  |  |
| **d.** | It will reduce the patient’s anxiety associated with uncertainty. |  |  |  |  |
| **e.** | To know when death is coming is an essential prerequisite for a good death. |  |  |  |  |
| **f.** | The family (not the doctor) should break the news to the patient. |  |  |  |  |

***On Advance Directives***

1. Are you aware of advance directives (living wills)? **(Select one response only)**
2. No, I have never heard of it
3. I have heard, but not well aware of it
4. I am well aware of it
5. Can an attempted suicide (deliberate self-harm) be considered as an advance refusal of life-saving treatment?

Yes

No

1. Would you transfuse blood in a patient in vascular shock due to active gastric bleeding and a haemoglobin of 4 g/dl, even if the patient has made an advance refusal of receiving any blood products?

Yes

No

***On withdrawal and withholding life sustaining treatment***

1. A 28-year-old doctor with metastatic carcinoma has developed respiratory failure. She could live for several weeks if she is placed on a ventilator. Would you place her on a ventilator?

Yes

No

1. A 28-year-old doctor who was ventilated following a road traffic accident has been confirmed to be brain dead. A 28-year-old man is in urgent need for a ventilator following deliberate self-harm with an insecticide. He could be saved if placed on a ventilator. There are no vacant ventilators available. Would you disconnect the doctor from the ventilator?

Yes

No

1. What time would you record as the ‘time of death’ in a brain dead patient who is disconnected from the ventilator?

....................................…................…………………………………………………………………………………………

1. Do you feel *more comfortable to* ***withhold*** *than to* withdraw life-sustaining therapy?

Yes

No

***On ‘Do not attempt cardiopulmonary resuscitation (DNACPR)’ decisions***

1. Are you aware of ‘do not attempt cardiopulmonary resuscitation (DNACPR)’ decisions?

**(Select one response only)**

1. No, I have never heard of it
2. I have heard, but not well aware of it
3. I am well aware of it

*If the answer is* ***‘a’*** *please skip question 11-15*

1. When would you consider a DNACPR order appropriate?
2. ………………………………………………………………………………………………………………………
3. ………………………………………………………………………………………………………………………
4. ……………………………………………………………………………………………………………………..
5. Who should make the DNACPR decision in an *unconscious* patient? **(select one response only)**
6. The medical team only
7. The family only
8. Both the medical team and family
9. Other (Please specify): ………………………………………………………………………………
10. Is it appropriate to withdraw all life sustaining therapy once a DNACPR decision has been made?

Yes

No

Don’t know

1. Would you feel reluctant to make a DNACPR decision on a patient?

Yes

No

*If yes*, why? ………………………………………………………………………………………………………………………………..

…………………………………………………………………………………………………………………………………………………….

Yes

No

1. Have you been involved in DNACPR decision?

***On the concept of a ‘Good Death’***

1. Once ‘dying’ (end-of-life) has been diagnosed, who should take the *lead* role in ensuring that the patient has a good death? **(Select one response only)**
   1. The caring physician
   2. The family
   3. A spiritual leader
   4. Nursing staff
   5. Other (Please specify):……………………………..
2. What would you consider as *essential characteristics of a good death*? **(Mark √)**

|  | **Yes** | **No** |
| --- | --- | --- |
| 1. To know when death is coming and to understand what can be expected |  |  |
| 1. To be able to retain control of what happens |  |  |
| 1. To be afforded dignity and privacy |  |  |
| 1. To have control over pain and other symptom control |  |  |
| 1. To have choice and control over where death occurs (at home or elsewhere) |  |  |
| 1. To have access to information and expertise of whatever kind is necessary |  |  |
| 1. To have access to any spiritual or emotional support required |  |  |
| 1. To have access to hospice care in any location, not only in hospital |  |  |
| 1. To have control over who is present and who shares the end |  |  |
| 1. To have time to say goodbye, and control over other aspects of timing |  |  |
| 1. To be able to leave when it is time to go, and not to have life prolonged pointlessly |  |  |
| 1. To have lived a long life |  |  |
| 1. To have lived a wholesome (virtuous) life |  |  |
| 1. To be able to issue advance directives which ensure wishes are respected |  |  |

***On medical ethics***

1. Name the four principles of medical ethics.
2. ……………………………………………………………….
3. ……………………………………………………………….
4. ……………………………………………………………….
5. ……………………………………………………………….
6. Should physician aid-in-dying (which includes both ‘physician-assisted suicide’ and ‘euthanasia’) be legalized in Sri Lanka for patients with incurable, progressive and painful disease? **(select one response only)**
7. Yes
8. No
9. Other (Please specify): ………………………………………..…………….
