# Supplementary material for: Knowledge and attitudes about end-of-life decisions, good death and principles of medical ethics among doctors in tertiary care hospitals in Sri Lanka: a cross-sectional study
Source: BMC Med Ethics. 2021 May 26;22:66. doi: 10.1186/s12910-021-00631-5 (PMC8152188; doi:10.1186/s12910-021-00631-5)
Supplement: Supplementary file 2 — Additional file 1: S2. Key to analysis. [file 12910_2021_631_MOESM2_ESM.docx]

**S2: Key to data analysis in the Questionnaire**

Knows that doctor should break bad news to patient **Q1**

Knows = responses (a) and (c).

Favours breaking bad news to terminally ill patient **Q2**

Favours = strongly agree or agree with responses (c), (d), (e) and/or strongly disagree or disagree with responses (a), (b), (f)

Adequately aware of advance directives **Q3**

Adequately aware = response (c)

Favours not placing a metastatic cancer patient on ventilator **Q6**

Favours = response ‘NO’

Favours disconnecting a brain-dead patient from ventilator **Q7**

Favours = response ‘YES’

Knows that time of death is the time of brain death **Q8**

Knows = response ‘time of death is the time of certifying brain death’

More comfortable withholding than withdrawing ventilation **Q9**

More comfortable = response ‘YES’

Adequately aware of DNACPR **Q10**

Adequately aware = response (c)

Aware when to consider DNACPR **Q11**

Aware = one or more of the following responses (1) when CPR is unlikely to succeed; (2) when the quality of life is likely to be worse after CPR; (3) in patients with progressive incurable disease; (4) when an advance directive has been made

Aware that the medical team makes the DNACPR decision in an unconscious patient **Q12**

Aware = response (a)

Knows that DNACPR does not entail withdrawing life-sustaining treatment **Q13**

Knows = response ‘NO’

No reluctance to make DNACPR decision **Q14**

No reluctance = response ‘NO’

The relevant question number in the Questionnaire is indicated in bold at the end of the statement.
